# Synthesis, Structure, Electrochemistry, and Spectral Characterization of Bis-Isatin Thiocarbohydrazone Metal Complexes and Their Antitumor Activity Against Ehrlich Ascites Carcinoma in Swiss Albino Mice

**DOI:** 10.1155/2008/362105

**Published:** 2007-11-19

**Authors:** M. P. Sathisha, V. K. Revankar, K. S. R. Pai

**Affiliations:** ^1^P. G. Department of Studies in Chemistry, Karnatak University, Dharwad 580 003, India; ^2^Department of Pharmacology, Manipal College of Pharmaceutical Sciences, Manipal Academy of Higher Education, Manipal 576 104, India

## Abstract

The synthesis, structure, electrochemistry, and biological studies of Co(II), Ni(II), Cu(II), and Zn(II) complexes of thiocarbohydrazone ligand are described. The ligand is synthesized starting from thiocarbohydrazide and isatin. It is evident from the IR data that in all the complexes, only one part of the ligand is coordinated to the metal ion resulting mononuclear complexes. The ligand coordinates essentially through the carbonyl oxygen of the isatin fragment, the nitrogen atom of the azomethine group, and sulfur atom after deprotonation to give five membered rings. H1 NMR spectrum of the ligand shows only one set of signals for the aromatic protons, while the NH of isatin and NH of hydrazone give rise to two different singlets in the 11–14 ppm range. The formulations, [Cu(L)Cl]·2${\text{H}}_{2}$O, [Cu(L)(C${\text{H}}_{3}$COO)]·2${\text{H}}_{2}$O, [Ni(L)Cl], [Ni(L)(C${\text{H}}_{3}$COO)], [Co(${\text{L}}_{2}$)], and [Zn(${\text{L}}_{2}$)]·2${\text{H}}_{2}$O are in accordance with elemental analyses, physical, and spectroscopic measurements. The complexes are soluble in organic solvents. Molar conductance values in DMF indicate the nonelectrolytic nature of the complexes. Copper complex displays quasireversible cyclic voltametric responses with *Ep* near −0.659 v and 0.504 v Vs Ag/AgCl at the scan rate of 0.1 V/s. Copper(II) complexes show a single line EPR signals. For the observed magnetic moment and electronic spectral data possible explanation has been discussed. From all the available data, the probable structures for the complexes have been proposed. The compounds synthesized in present study have shown promising cytotoxic activity when screened using the in vitro method and at the same time were shown to have good activity when tested using the Ehrlich ascites carcinoma (EAC) model. The antimicrobial screening showed that the cobalt complex possesses enhanced antimicrobial activity towards fungi.

## 1. INTRODUCTION

Although ligands having oxygen and nitrogen as donor atoms are by far the most extensively studied, interest in sulfur donor chelating agents has grown over the years and the number of chemical studies in this area has increased considerably [1]. Interest in complexes of these ligand systems now covers several areas, ranging from general considerations of the effect of sulfur and electron delocalization in transition metal complexes to potential biological activity and practical application [2–4].

**Figure 1:**
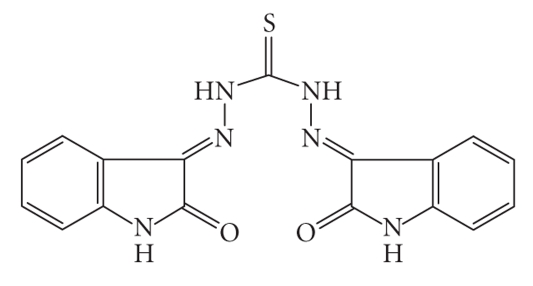
Representative structure of the ligand.

The aim of the present work is to synthesize new thiocarbohydrazone ligand, and to study its coordination behavior with Co(II), Ni(II), Cu(II), and Zn(II) ions. N, ${\text{N}}^{\prime }$-Thiocarbohydrazide condenses easily with two molecules of carbonyl compounds *viz*., isatin, on the N^1^H_2_ and N^4^H_2_ hydrazine amino groups to produce the desired ligand shown in Figure 1. The tautomerism in this ligand and also the well-known tendency of oxygen and sulfur donors to act as bridging sites [5, 6] allows various structural possibilities for the corresponding metal complexes.

A considerable number of metal complexes are now known to possess antitumor activity [7]. There are reports that sulphur-containing ligands [8] and platinum complexes of sulphur-containing amino acids were found to have inhibitory action against tumors [9]. However, since the class of sulphur-containing compounds such as thiosemicarbazones, an structural analogue of the thiocarbohydrazone, have been reported to have anticancer activity owing to the specific and unique properties of their metal chelate [10], it is suggested that the present compounds, being similar in structure and character, may be acting by virtue of their chelating properties at the cellular level thereby exerting their anticancer activity. Hence, it was considered worthwhile to subject the presently studied complexes for evaluation of their anticancer activity.

The antimicrobial properties of metals have been recognized for centuries and have represented some of the most fundamental breakthroughs in medicinal history [11]. Many studies stressed the role of metal ions in important biological processes, whereas the inorganic pharmacology started to be an important field with more than 25 inorganic compounds, being used in therapy as antibacterial, antiviral, and anticancer drugs [12, 13]. Kirschner et al. [14] have suggested that the transfer of the metal ion from the ligand to the cancer-associated viruses was an important mechanism for designing new anticancer therapies. The inverse process, that is, coordinating a metal ion from an important biomolecule, such as, for instance, a zinc finger protein, has recently been used to design novel antiviral therapies, targeted against human immunodeficiency (HIV) and human papilloma virus (HPV) infections [15]. We have already drawn attention to the strong relationship between metals or their complexes [16–18], and antibacterial [19], antitumour [20], and anticancer [21] activities. A number of in vivo studies have indicated [22] that biologically active compounds become more bacteriostatic and carcinostatic upon chelation.

## 2. EXPERIMENTAL

### 2.1. Materials, analytical methods, and physical measurements

All chemicals used were of reagent grade. Solvents were distilled prior to use. The metal content of the complexes were estimated after decomposition with mixture of HCl and HClO_4_ by gravimetric method (copper and nickel) and EDTA titration method (cobalt and zinc). Magnetic susceptibility of complexes were measured at room temperature on a Faraday balance using Hg[Co(SCN)_4_] as a calibrant. Electronic spectra were recorded using VARIAN CARY 50 Bio UV-Visible spectrophotometer in DMSO. The IR spectra of ligand and its complexes were recorded as KBr pellets in the region 4000–400 cm^−1^ on Nicolet 170 SX FT-IR spectrometer. The ^1^H-NMR spectra of ligand and its zinc (II) complex were recorded in DMSO-d_6_ on Bruker 300 MHz spectrometer using TMS as an internal standard. The cyclic voltammetry experiments were carried out with a three-electrode apparatus using a CHI1110A electrochemical analyzer (USA). The EPR spectra of copper (II) complexes were recorded at room temperature on Varian E-4 X-band spectrometer using TCNE as *g*-marker. Conductivity measurements were made on 10^−3^ M solutions of complexes in DMSO using ELICO-CM82 conductivity bridge provided with a cell having cell constant 0.51.

### 2.2. Synthesis of N,${\text{N}}^{\prime }$-Thiocarbohydrazone bis (isatin)

**Scheme 1:**
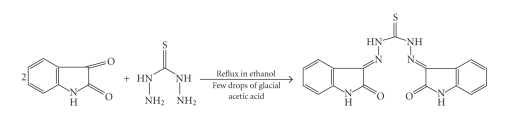
Synthesis of ligand.

A solution of isatin 5.8 g (0.04 mol) in ethanol (30 ml) was added drop wise to a refluxing solution of thiocarbohydrazide 2.1 g (0.02 mol) in the same solvent (40 ml). Few drops of glacial acetic acid were added to the reaction mixture and was refluxed for 2 hours. At the end of the reaction, monitored by TLC (n-hexane/ethyl acetate 2:3), a yellow-orange powder was filtered and washed with warm ethanol and then with diethyl ether. Yield 75 %. M.P. 284°C (Scheme 1).

The thiocarbohydrazide was prepared as described in the literature [23].

### 2.3. Synthesis of metal (II) chloride and acetate complexes

**Scheme 2:**
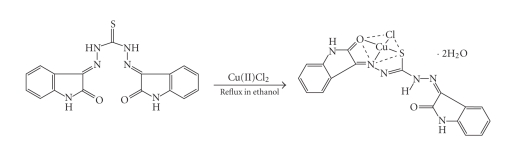
Representative synthesis of copper chloride complex.

In a representative preparation, the complex was prepared by the addition of ethanolic solution of metal (II) chloride or aqueous ethanolic solution of metal (II) acetates (0.003 mol) with constant stirring to the corresponding amount of the ligand (0.003 mol) in the same solvent. The mixture was heated to reflux for 1 hour. The product was filtered off, washed several times with ethanol, and dried in vacuum over P_2_O_5_ (Scheme 2).

### 2.4. Brine shrimp lethality bioassay [24]

The brine shrimp lethality test was used to predict the presence of cytotoxic activity. The brine shrimp (Artemia salina) eggs were procured from www.brineshrimp.com. The brine shrimp eggs were hatched in artificial sea water at room temperature under constant aeration for 48 hours. After hatching, 10 larvae were placed in a vial containing 5 ml of artificial sea water. A drop of dry yeast suspension (3 mg in 5 ml sea water) was added to each vial as food for shrimps. Test compounds in different concentrations (10, 100, and 1000 ppm) were added to the vials before making up the final volume to 5 ml with sea water. The control group had shrimp in artificial sea water. The vials were maintained under illumination with 40 W electric bulb. The experiments were done in triplicate and mean of three readings was taken as final result. After 24 hours, the survivors were counted using a 3X magnifying glass, and the percent deaths and LC_50_ (Lethal concentration for half the population) values wear calculated by using Finney computer program.

### 2.5. Cell lines

Cancer cell lines viz. Ehrlich ascitic carcinoma (EAC), to induce cancer in animal model (mice) were obtained from (Amala Cancer Research Center, Amala Nagar, Kerala, India.) The cells were maintained as ascites tumor in Swiss Albino mice by intraperitoneal inoculation of $1\times 10^{6}$ viable cells.

### 2.6. Animals

Six-to-eight week old female Swiss Albino mice (25±5 g body weight) were selected from (Central Animal Facility, Manipal Academy of Higher Education, Manipal, Karnataka, India.) The animals were acclimatized to the experimental room having temperature $23\pm 2^{{}^{\circ}}$C, controlled humidity conditions, and 12:12 hour light and dark cycle. The mice were housed in sterile polypropylene cages containing sterile paddy husk as bedding material with a maximum of 4 animals in each cage. The mice were fed on autoclaved standard mice food pellets (Hindustan Lever) and had access to water *ad libitum*. The animal experiments were performed according to the rules and regulations of the Institutional Animal Ethics Committee (IAEC).

### 2.7. Preparation of test solution of compounds

The compounds (dose 50 mg/kg), suspended in 4 % gum acacia (gummy exudates from the bark of Acacia senegal, an inactive substance, which forms a mucilage with water) were administrated intraperitoneally, daily once for 5 days from day 10 posttumor inoculation in a volume of 0.1 ml/10 g mouse. The dose of cisplatin selected was 3.5 mg/kg. This was calculated from the human dose using an appropriate conversion factor [25].

## 3. ANTITUMOR ACTIVITY

### 3.1. Determination of cytotoxicity of compounds to EAC cells (*in vitro* studies) Tryphan blue exclusion method (cell viability test)

In vitro short-term cytotoxic activity of drug was determined using EAC cells. The EAC cells that were collected from the animal peritoneum by aspiration were washed repeatedly with phosphate buffered saline (PBS) to free it from blood. The viability of the cells was checked in a haemocytometer. The cells ($1\times 10^{6}$ in 0.1 ml PBS) were incubated in clean sterile tubes with the test compounds (0.01 ml, 1–50 μg/ml in dimethyl sulfoxide (DMSO)) for 3 hours at 37°C, keeping the final volume at 0.9 ml. The volume of DMSO was pegged below 0.1 % of the total volume. The control tube had 10 μl of solvent. The final volume was made up to 0.9 ml with PBS. To each tube 100 μl of Tryphan blue solution was added. The live (without stain) and dead (with blue stain) cells were counted using haemocytometer and percent cell death was calculated using the formula:

(1) $$\%\text{Cytotoxicity}=100\times ({\text{T}}_{\text{dead}}-{\text{C}}_{\text{dead}})/{\text{T}}_{\text{tot}},$$

where ${\text{T}}_{\text{dead}}$ is the number of dead cells in the treated group, ${\text{C}}_{\text{dead}}$ is that in the control group, and ${\text{T}}_{\text{tot}}$ is the total number of dead and live cells in the test compound treated group. Cisplatin was used as the standard [26].

### 3.2. Induction of Ehrlich Ascites Carcinoma [27]

Antitumor activity of the compounds was determined using Ehrlich ascites carcinoma (EAC) tumor model in mice. Female Swiss Albino mice were divided into groups of 12 animals each. ((a) Normal mice for hematological studies, (b) Tumor-bearing mice, (c) Tumor-bearing mice treated with one dose of cisplatin, (d) Tumor-bearing mice groups treated with compounds for 5 days.). The ascitic carcinoma-bearing mice (donor) were used for the study, 15 days after tumor transplantation. The ascitic fluid was drawn using an 18-gauge needle into sterile syringe. A small amount was tested for microbial contamination. Tumor viability was determined by Tryphan blue exclusion test and cells were counted using haemocytometer. The ascitic fluid was suitably diluted in normal saline to get a concentration of 10^6^ cells/ml of tumor cell suspension. This was injected intraperitoneally to obtain ascitic tumor. The mice were weighed on the day of tumor inoculation and then once in three days thereafter. Treatment was started on the tenth day of tumor inoculation. Cisplatin (one dose) was injected on tenth day intraperitoneally. The compounds were administered from tenth day for 5 days intraperitoneally. After the administration of last dose followed by 18-hour fasting, six mice from each group were sacrificed for the study of antitumor activity and hematological parameters. The remaining animals in each of the groups were kept to check the mean survival time (MST) of the tumor-bearing hosts. Antitumor effects of compounds were assessed by observation of following parameters.

### 3.3. Percentage increase in weight as compared to day-0 weight

Upon weighing the animal on the day of inoculation and after once in three days in the postinoculation period, the percentage increase in weight was calculated using the formula: % Increase in weight = [(animal weight on respective day/animal weight on day-0) −1] ×100 [28].

### 3.4. Median survival time and increase in lifespan [% ILS]

Total number of days an animal survived from the day of tumor inoculation was counted. Subsequently, the median and mean survival time were calculated. The percentage increase in lifespan (% ILS) was calculated using the formula: ILS (%) = [(mean survival time of treated group/mean survival time of control group) −1]×100 [26].

### 3.5. Hematological parameters [29]

In order to detect the influence of compounds on the hematological status of EAC-bearing mice, comparison was made amongst groups of mice for each compound on the fourteenth day after transplantation. Blood was drawn from each mouse from retro orbital under ether anesthesia and the white blood cell (WBC) total count, differential leukocyte counts, red blood cell (RBC) total count, and hemoglobin content parameters were evaluated.

### 3.6. Statistical analysis

Results were analyzed by one-way ANOVA by Scheffe's posthoc test using SPSS computer package.

## 4. EVALUATION OF ANTIBACTERIAL AND ANTIFUNGAL ACTIVITIES

### 4.1. Antibacterial activity

Antibacterial activity of test compounds was assessed against *Bacillus cirroflgellosus* by cup-plate method [30].

#### 4.1.1. Materials

1. Nutrient agar.
2. Sterilized petridishes and pipettes.
3. 20–24-hour old subcultures in nutrient agar medium.
4. Sterilized test tubes containing solution of the test compounds in desired concentration.

#### 4.1.2. Preparation of inoculation medium

The definite volumes of peptone (0.5 %), yeast extract (0.15 %), beef extract (0.15 %), sodium chloride (0.35 %), dipotassium phosphate (0.36 %), and potassium dihydrogen phosphate (0.13 %) were dissolved in distilled water and the *p*H was adjusted to 7.2. This solution was sterilized by autoclaving at 15 p.s.i. for 20 minutes.

#### 4.1.3. Preparation of subcultures

One day prior to these tests, inoculation of above bacterial cultures was made in the inoculation medium as described above and incubated at 37°C for 18–24 hours.

#### 4.1.4. Preparation of base-layer medium

Base-layer medium was prepared by dissolving definite volumes of peptone (0.6 %), yeast extract (0.3 %), beef extract (0.13 %), and agar (2.5 %) in distilled water. The *p*H of this medium was also adjusted to 7.2 and sterilized by autoclaving at 15 p.s.i. for 20 minutes.

#### 4.1.5. Preparation of test compounds

Each test compounds (5 mg) was dissolved in dimethylformamide (5 ml) to give a solution of 1000 μg/ml. Out of this 0.1 ml. of solution was used for antimicrobial testing.

#### 4.1.6. Testing method

Base-layer was obtained by pouring about 10–15 ml of base-layer medium into each sterilized petridishes and were allowed to attain room temperature. This solid layer after attaining room temperature is called base-layer. Over-night grown sub-cultures of bacteria were mixed with seed layer medium and immediately poured into petridishes containing the base-layer and then allowed to attain room temperature.

The cups were made by scooping out nutrient agar with a sterile cork borer. To these cups, solutions of test compounds (0.1 ml) were added using sterile pipettes and these plates were subsequently incubated at 37°C for 36 hours. The zone of inhibitions, if any, was measured in mm for the particular compound. Norfloxacin was used as positive-control and solvent-control was also used to know the activity of the solvent. The results of antibacterial testing are summarized in Table 8.

### 4.2. Antifungal activity

Fungicidal activity of test compounds was assessed against *Aspergillus niger* and *Candida albicans* by cup-plate method.

#### 4.2.1. Materials

1. Nutrient agar.
2. Sterilized Petri dishes and pipettes.
3. 16–18 hours old sub-cultures in nutrient agar medium supplemented with 1% glucose.
4. Sterilized test tubes containing solutions of the compounds in desired concentration.

#### 4.2.2. Preparation of inoculation medium

Inoculation medium was prepared by dissolving definite volumes of peptone (1.0 %), yeast extract (0.6 %), sodium chloride (0.5 %), potassium dihydrogen phosphate (0.3 %), and glucose (1.0 %) in distilled water. The *p*H of the medium was adjusted to 6.0 and sterilized at 15 p.s.i. for 20 minutes.

#### 4.2.3. Preparation of sub-cultures

One day before testing, inoculation of fungi, were made in the inoculation medium and incubated at 37°C for 18–24 hours.

#### 4.2.4. Preparation of base-layer medium

The definite volumes of peptone (4.0 %), yeast extract (0.6 %), sodium chloride (0.5 %), potassium dihydrogen phosphate (0.3 %), glucose (1.0 %), and agar (2.5 %) were dissolved in distilled water. The *p*H of the medium was adjusted to 6.0 and sterilized by autoclaving at 15 p.s.i. for 20 minutes.

#### 4.2.5. Preparation of seed layer medium

The definite volumes of peptone (4.0 %), yeast extract (0.6 %), sodium Chloride (0.5 %), potassium dihydrogen phosphate (0.3 %), glucose (1.0 %) and agar (2.5 %) were dissolved in distilled water. The *p*H of medium was adjusted to 6.0 and was sterilized separately by autoclaving at 15 p.s.i. for 20 minutes.

#### 4.2.6. Testing method

The method of testing for antifungal activity is the same as that adopted for assessing antibacterial activity. Grisofulvin was used as a positive control and solvent content was also used to know the activity of the solvent. The fungicidal results are summarized in the Table 8.

## 5. RESULT AND DISCUSSION

The ligand was synthesized by acid catalysed condensation of thiocarbohydrazide with the isatin in ethanol (Scheme 1); the condensation proceeds, as usual, selectively on the carbonyl in position 3 in the isatin ring. The ligand is characterized by means of IR, ^1^H NMR spectroscopy, and elemental analysis. In the ^1^H NMR spectrum of the ligand there is only one set of signals for the aromatic protons, while the NH of isatin and NH of hydrazone give rise to two different singlets in the 11–14 ppm range. The presence of the NH groups is confirmed in the IR spectra by a broad peak around 3200 cm^−1^; the C=O groups absorb near 1690 cm^−1^.

The reactions between the metal(II) chlorides/acetates and the ligand (1:1) lead to formation of complexes, (Scheme 1). The growth of single crystals of these complexes for X-ray studies is very difficult owing to their amorphous nature and we were unsuccessful in our attempts to do so. The elemental analyses of these complexes reveal 1:1 ligands to metal stoichiometry in case of Cu(II) and Ni(II). Copper forms the complexes of the type [Cu(L)Cl]⋅2H_2_O with Cu(II) chloride and [Cu(L)OAc]⋅2H_2_O in case of Cu(II) acetate. Nickel forms [Ni(L)Cl] type complex with Ni(II) chloride and [Ni(L)OAc] with acetate. Both cobalt and zinc chloride and acetates form the same complexes with the 2:1 ligand to metal stoichiometry, with general formula, [M(L_2_)]. The complexes are found to be soluble in dimethylformamide, dimethylsulphoxide, and acetonitrile but insoluble in common organic solvents such as ethanol, methanol, benzene, acetone, and so forth. The composition and coordination geometry of these complexes has been established on the following experimental observations. The molar conductance values in dimethylsulphoxide fall in the expected range (10–32 cm^2^Ω^−1^mol^−1^) of nonelectrolytes [31]. The complexes were analyzed for metal, nitrogen, carbon, hydrogen and chloride. The analytical, conductivity, and magnetic moment data of the complexes are summarized in Table 1.

### 5.1. Infrared spectral studies

The IR spectral data of the ligand N, ${\text{N}}^{\prime }$-*bis*(isatin)thiocarbohydrazone shows a sharp absorption band around 3200 cm^−1^ attributed to the presence of NH group and a very strong band near 1690 cm^−1^ assigned to (C=O) stretching vibration. The band at 1619 cm^−1^ is assigned to the azomethine (C=N) stretching. The Ir spectral assignment of metal complexes was aided by comparison with the vibration frequencies of the free ligand. The broad band that appears in the range of 3160–3210 cm^−1^ is assigned to the stretching vibration of ring (N−H). There is only one band in the v(C=O) region and it does not differ significantly from the band in the ligand; suggesting that the carbonyl groups are weakly involved in the coordination. Although there is coordination of nitrogen atom of the azomethine group to the central metal atom, we could not appreciably conclude from the Ir data, because of remaining uncoordinated azomethine group that absorbs at a just lower frequency near 1614-1615 cm^−1^ as compared to their ligand [32]. The band corresponding to the stretching vibration of the C=S group appears at 1200–1194 cm^−1^ in the ligand [33]. The absence of this band in the Ir spectra of the metal complexes can be explained by the tautomerism of the C=S group with one of the imino groups to form the C−SH and the coordination of sulphur after deprotonation. The band that appears in the range of 690–750 cm^−1^ is thus assigned to the ν (C−S) in the Ir spectra of the metal complexes. The Ir spectra of the complexes derived from copper and nickel acetate, show an absorption bands in the region 1665–1650 cm^−1^ which is assigned to $\nu_{8}$ (C−O) antisymmtric stretching of acetate group and another in the region 1297–1258 cm^−1^ and which can be assigned to $\nu_{3}$ (C−O) symmetric stretching vibration of acetate. Difference $\nu_{8}-\nu_{3}$ which is around 407–493 cm^−1^ indicates the unidentate coordination of the acetate group [34]. The complexes show a broad band around 3400 cm^−1^, which is assigned to the ν(H_2_O) absorption. The ν(M−N) and ν(M−O) stretching vibrations are observed at about 490 and 455 cm^−1^, respectively, in the spectra of the complexes [35].

### 5.2. ^1^H NMR spectral studies

The ^1^H NMR spectrum of the ligand shows only one set of signals for the aromatic protons around 6.93–7.60 ppm, while the NH of isatin and NH of hydrazone give rise to two different singlets in the 11–14 ppm range.

The ^1^H NMR spectrum of the Zn(II) complex is less informative, the presence of the hydrazonic proton is confirmed by a peak at about 13 ppm, which is slightly shifted to down field after complexation. Peak due to ring NH and remaining signals of the ligand remain substantially unchanged upon complexation.

### 5.3. Electronic spectral studies

Electronic spectral data of the ligand and their transition metal complexes were recorded in DMF solutions. In the electronic spectrum of the ligand, three prominent absorption bands at 266, 382, and 670 nm were characterized. The band at 266 nm corresponds to the $\pi \to \pi^{*}$ transition of the C=S group [36]. The band at 382 nm corresponds to the transition of azomethine group and the band at 670 nm corresponds to the $n\to \pi^{*}$ transition from the amide oxygen of the isatin moiety to the azomethine group [37].

In case of complexes, the bands appeared in the almost same position as they were appeared in ligand. Followed by these, the bands displayed at 485, 485 and 495 nm in case of Cu(II), Ni(II) and Co(II) complexes respectively are assigned to ligand to metal charge transfer transitions (LMCT). Apart from these, the bands around 899, 780 nm in case of Cu(II) and Ni(II) complexes were assigned as d-d transition bands.

### 5.4. Cyclic voltametric studies

The electrochemical behavior of copper(II) complex has been investigated in DMSO containing 0.1 M tetraethyl ammonium chloride supporting electrolyte, using glassy carbon working electrode and Ag/AgCl, Pt electrodes as reference and counter electrodes, respectively. The complex involves single redox step corresponding to Cu(II)→Cu(I), quasireversible electrode process at *Epc* =−0.659 V and the associated anodic peak at *Epa* =0.504 V [38] suggesting the tetrahedral environment of the copper ion.

### 5.5. Magnetic measurement studies

The magnetic moments of the complexes were recorded at room temperature and the observed magnetic moment value for the Co(II) complex is 4.98 BM, which is in the range of 4.4 to 5.5 BM observed for the octahedral Co(II) complexes.

The Ni(II) complexes derived from chloride and acetate salts exhibit the magnetic moment values 3.55 and 2.92 BM suggesting tetrahedral environment around metal ion. The value of 1.91 BM for Cu(II) chloride and 1.89 BM for Cu(II) acetate complex suggests the four coordinated tetrahedral complexes, which further supported by their electronic spectral data and CV studies.

### 5.6. ESR spectral studies

X-band ESR spectra of the polycrystalline Cu(II) complex were recorded at room temperature. The ESR spectra of the mononuclear copper chloride and acetate complexes show a $g_{\text{iso}}$ value in the range of 2.066 and 2.092 respectively.

### 5.7. Antitumor activity of complexes against Ehrlich ascites carcinoma in Swiss Albino mice.

#### 5.7.1. Brine shrimp lethality bioassay

The Brine shrimp lethality bioassay has been chosen to assess the in vitro cytotoxic effects of the compounds, as it is an inexpensive, reliable, and quick method for the purpose [24]. All the tested compounds showed considerable cytotoxic activity in the brine shrimp lethality bioassay. LC_50_ concentrations for the compounds are tabulated in Table 2.

#### 5.7.2. Tryphan blue exclusion method (cell viability test)

The compounds were tested using the short-term in vitro cytotoxicity towards EAC cells as a preliminary screening technique of Tryphan blue exclusion method (cell viability test) for their cytotoxic potential [26]. This is one of the methods to assess cytotoxicity of anticancer compounds. This test is based on the principle that living cell membrane has the ability to prevent the entry of dye. Hence, they remain unstained and can be easily distinguished from dead cells, which take the dye. The percentage of viable cells was determined. Results of the short-term in vitro cytotoxicity of the compounds are shown in Table 3. These preliminary experiments were carried out mainly with five different concentrations of the compounds. All the compounds were found to be cytotoxic and produced 50% cell death at or below a concentration 19.1 μg/ml. At 50 μg/ml concentration, the standard (cisplatin) showed 98 % cell death. At 50 μg/ml concentration, the [Cu(L)OAc]⋅2H_2_O, [Cu(L)Cl]⋅2H_2_O showed more than 80 % cell death. All the compounds were found to have considerable cytotoxicity in the cell viability test.

#### 5.7.3. Anticancer studies in Ehrlich Ascites Carcinoma method

**Weight variation parameter**

The tumor inoculated control animals recorded significant weight gain by day-0. They gained a maximum weight of 19 % by day-15. Cisplatin administration (on tenth postinoculation day) significantly (P<.05) reduced weight gain as compared to control on day-15. The compounds also significantly (P<.05) reduced the weight gain on day-15 as compared to control (Table 4). Comparative effects of treatments versus cisplatin on reduction of body weights in tumor-induced mice are given in Table 5.

**Survival time parameter**

The effect of compounds on survival of tumor-bearing mice is shown in Table 6. Cisplatin significantly prolonged the median and mean survival times (P<.05) with respect to its control. It showed a significant increase in the percentage lifespan of animals (ILS >50). On the other hand, all the compounds significantly prolonged the mean survival Times. The influence of all the compounds on % ILS was more than 25 %. By convention, a 25 % increase in lifespan is considered as possible anticancer activity of a test compound [39].

**Hematological parameters**

The effect of compounds on hematological parameters is shown in Table 7. Tumor induction significantly (P<.05) increased the total number of WBC by almost 4 times. Cisplatin administration reversed this effect significantly (P<.05). All the compounds significantly (P<.05) reversed the tumor-induced rise in total counts of WBC. However, they were not as efficacious as cisplatin in reversing the tumor-induced total counts. On differential counts, tumor-induction caused a significant reduction in lymphocyte and a significant (P<.05) increase in neutrophil counts. This was significantly (P<.05) reversed towards normal by cisplatin and the test compounds. However, the compounds were less efficacious than cisplatin in their effects. Tumor induction caused significant decrease in RBC and Hb almost to the half of the normal animals [40]. This was significantly (P<.05) reversed towards normal by cisplatin and the test compounds.

### 5.8. Antimicrobial studies

In the light of interesting antimicrobial activities of the coordination complexes, the ligands and their corresponding complexes were screened for antifungal and antibacterial activity against *Aspergillus niger, Candida albicans*, and *Bacillus cirroflagellosus*, respectively, by the cup plate method using Nutrient agar. The radial growth of the colony was recorded on completion of the incubation and the mean diameter for each complex at a single concentration was recorded. The average percentage inhibition of the fungicidal growth medium compared using the Vincent equation [41]: I=100(C−T)/C, where I= percentage inhibition, T = average diameter of the fungal and bacterial growth on the tested plates, and C= average diameter of the growth on the control plates. The screening data of the inhibition of the fungi and bacteria are given in Table 8. From the data, it is clear that the free ligand N, ${\text{N}}^{\prime }$-*bis*(isatin)thiocarbohydrazone is moderately active against *B. cirroflagellosus* where as highly active against fungi *A. niger* and *C. albicans*. On complexation, there is a notable enhancement of both antibacterial as well as antifungal activity. The promising results were observed for the Co(II) complex against both the fungi. The higher fungi toxicity exhibited by the complexes may be ascribed to the fact that the metal complexes are more susceptible to fungal cells than bacterial cells.

## 6. CONCLUSION

**Figure 3:**
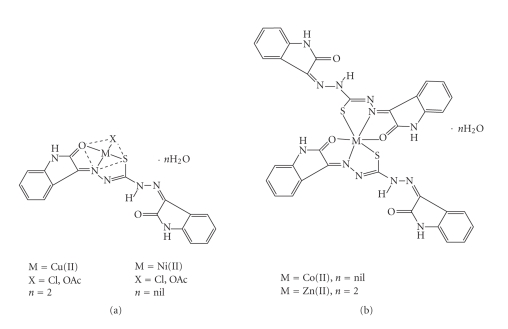
Proposed structures of the complexes.

From the elemental analysis, molar conductivity, UV-Visible, magnetic, IR and ^1^H NMR spectral data it was possible to determine the type of coordination of the ligand in their metal complexes. In all the complexes, only one part of the ligand is coordinated to the metal ion resulting mononuclear complexes. The ligand coordinates essentially through the carbonyl oxygen of the isatin fragment, the nitrogen atom of the azomethine group and sulfur atom after deprotonation to give five membered rings. The ligand acts as a monobasic, tridentate (Figure 3).

The compounds showed considerable cytotoxic activity in the trypan blue exclusion method. In the in vivo cancer model (Ehrlich ascitic carcinoma model), the compounds significantly (P<.05) reversed the tumor-induced changes in the parameters monitored viz., percentage increase in body weight, percentage increase in lifespan, tumor-viable count, and hematological parameters (total and DLC of WBC, total RBC, and Hemoglobin count). These effects were almost comparable to cisplatin—the standard drug used in the study. The compounds, however, were found to have good effect in prolonging the lifespan (ILS) as compared to standard drug cisplatin. These findings imply that the compounds might be having some anticancer principles.

Based on the data of the present study, it is very difficult to suggest the possible mechanism of these compounds for anticancer effects. The compounds tested in our present study have shown promising cytotoxic activity when screened using the in vitro method and at the same time were shown to have good activity when tested using the Ehrlich ascites carcinoma model. Though it is very difficult to conclude anything at this stage, it can be assumed that after testing against various other cancer models and at different doses these compounds may prove to be safer drugs for tomorrow.

Further, the promising results were observed for the antimicrobial screening especially for the Co(II) complex against both the fungi and what may be attributed to the fact that the metal complexes are potentially active against fungal cells than bacterial cells.

## Figures and Tables

**Figure 2:**
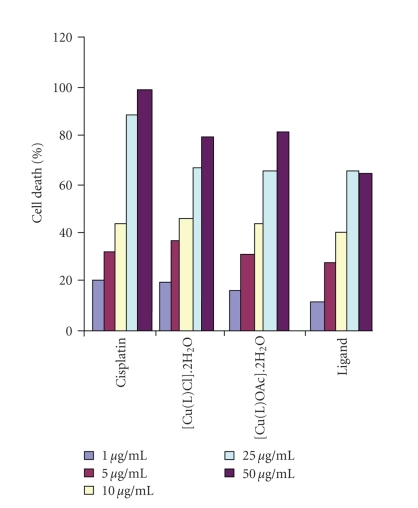
Short-term in vitro cytotoxicity of compounds towards EAC cells.

**Table 1:** Elemental analysis, molar conductance, and magnetic moment measurements.

Compounds	Found (Calc.) %					Molar conductance	Magnetic moment BM
	C	H	N	M	CI	$\lambda_{m}$(cm^−1^ $\Omega^{-1}$mol^−1^)	
Ligand	55.8(56.1)	2.8(3.2)	23.0(23.1)	—	—	—	—
[Co(L_2_)]	50.9(51.1)	2.8(2.2)	21.4(20.7)	7.2(7.5)	—	2.1	4.98
[Ni(L)Cl]	43.5(44.7)	2.0(2.4)	17.9(18.4)	12.2(12.8)	7.3(7.7)	3.5	3.55
[Ni(L) OAc]	40.3(41.8)	2.3(2.8)	15.7(16.2)	10.9(11.4)	—	2.6	2.92
[Cu(L)Cl]⋅2H_2_O	39.6(40.8)	2.3(2.8)	16.1(16.8)	11.9(12.7)	6.7(7.1)	2.2	1.91
[Cu(L)OAc]⋅2H_2_O	43.1(43.6)	2.9(3.4)	15.9(16.1)	11.8(12.2)	—	2.8	1.89
[Zn(L_2_)]⋅2H_2_O	46.8(47.2)	1.9(2.5)	18.6(19.4)	7.1(7.5)	—	0.7	—

**Table 2:** Brine shrimp bioassay results of compounds.

SL. no	Compound	Percentage deaths at 24 hr			LC_50_ mg/ml
		5 mg/ml	10 mg/ml	20 mg/ml	
1	[Cu(L)Cl]⋅2H_2_O	40	72	100	6.17
2	[Cu(L)OAc]⋅2H_2_O	27.27	66.66	100	7.26
3	Ligand	9.1	25	90	11.83

**Table 3:** Short-term in vitro cytotoxicity of compounds towards EAC cells.

Compounds	Percentage cell-death at different concentrations after 3 hours					LC_50_ μg/mL
	1 μg/mL	5 μg/mL	10 μg/mL	25 μg/mL	50 μg/mL	
Cisplatin	20	32	44	88	98	6.6377
[Cu(L)Cl]⋅2H_2_O	19	37	46	67	79	11.1475
[Cu(L)OAc]⋅2H_2_O	16	31	43	65	82	10.9732
Ligand	12	28	40	56	64	19.1815

**Table 4:** Effect of drugs on body weight changes in tumor-induced mice.

Group	Dose (mg/Kg) i.p.	% increase in weight as compared to Day-0 (mean ± SE)				
		Day-3	Day-6	Day-9	Day-12	Day-15
Control	—	4.46±1.98	7.04±2.19	16.13±1.75	17.15±1.89	19.15±2.22
Cisplatin	3.5	4.51±1.15	12.04±1.38	19.16±1.34	${9.91\pm 2.12}^{\text{a}}$	${1.32\pm 4.23}^{\text{a}}$
[Cu(L)Cl]⋅2H_2_O	50	5.45±1.25	16.27±3.29	22.13±5.76	15.35±1.88	${4.56\pm 2.56}^{\text{a}}$
[Cu(L)OAc]⋅2H_2_O	50	5.55±1.83	14.19±2.37	21.09±2.42	15.24±2.65	${6.36\pm 2.55}^{\text{a}}$
Ligand	50	4.88±2.02	14.33±4.37	24.69±4.99	16.54±2.35	${7.76\pm 3.43}^{\text{a}}$

${}^{\text{a}}$ P<.05 versus Control.

**Table 5:** Comparative effects of treatments versus cisplatin on reduction of body weights in tumor-induced mice.

Group	Dose (mg/kg) i.p.	% Decrease in weight as compared to respective control (Mean ± SE)	
		Day 12 (Mean ± SE)	Day 15 (Mean ± SE)
Cisplatin	3.5	42.22±11.32	93.10±12.31
[Cu(L)Cl]⋅2H_2_O	50	10.49±9.31	76.19±10.31
[Cu(L)OAc]⋅2H_2_O	50	11.14±6.43	66.79±12.70
Ligand	50	3.56±9.28	59.48±5.68

**Table 6:** Effect of drugs on the survival time in tumor-induced mice.

Group	Dose (mg/kg)	Median survival time (days)			Mean survival time (days)		
		MST	%T/C	%ILS	(Mean ± SEM)	%T/C	%ILS
Control	—	18.00	—	—	18.33±0.21	—	—
Cisplatin	3.5	34.50	191.67	91.67	${34.33\pm 0.33}^{\text{a}}$	187.28	87.28
[Cu(L)Cl]⋅2H_2_O	50	26.00	144.44	44.44	${26.17\pm 0.31}^{\text{a}}$	142.77	42.77
[Cu(L)OAc]⋅2H_2_O	50	25.00	138.88	38.88	${24.67\pm 0.21}^{\text{a}}$	134.59	34.59
Ligand	50	23.00	127.77	27.77	${23.00\pm 0.37}^{\text{a}}$	125.48	25.48

${}^{\text{a}}$ P<.05 versus control groups, MST = Median Survival Time.

**Table 7:** Effect of the compounds on hematological parameters.

Group	Dose	RBC	Hb	WBC	Differential Leucocyte Count %		
	(mg/kg)	(Mean ± SE)	(Mean ± SE)	(Mean ± SE)			
		(Millions/mm^3^)	(g%)	(10^3^cells/ mm^3^)	Lymphocytes	Neutrophils	Monocytes
Normal	—	5.03±0.28	15.72±0.32	8.59±0.16	86.23±0.61	13.03±0.46	1.07±0.33
Control	—	${2.14\pm 0.34}^{\text{a,c}}$	${9.53\pm 0.32}^{\text{a,c}}$	${32.31\pm 0.67}^{\text{a,c}}$	${41.80\pm 0.39}^{\text{a,c}}$	${57.35\pm 0.61}^{\text{a,c}}$	1.25±0.43
Cisplatin	3.5	${3.26\pm 0.24}^{\text{a,b}}$	${13.08\pm 0.29}^{\text{a,b}}$	${10.90\pm 0.19}^{\text{a,b}}$	${77.97\pm 0.48}^{\text{a,b}}$	${20.80\pm 0.47}^{\text{b}}$	1.22±0.54
[Cu(L)Cl]⋅2H_2_O	50	${3.84\pm 0.51}^{\text{a,b,c}}$	${11.71\pm 0.29}^{\text{a,b,c}}$	${17.84\pm 0.31}^{\text{a,b,c}}$	${68.97\pm 0.20}^{\text{a,b,c}}$	${29.72\pm 0.22}^{\text{a,b}}$	1.32±0.31
[Cu(L)OAc]⋅2H_2_O	50	${4.49\pm 0.30}^{\text{a,b,c}}$	${12.79\pm 0.17}^{\text{a,b,c}}$	${19.27\pm 0.99}^{\text{a,b,c}}$	${68.10\pm 0.45}^{\text{a,b,c}}$	${30.55\pm 0.47}^{\text{a,b}}$	1.35±0.43
Ligand	50	${4.72\pm 0.11}^{\text{a,b,c}}$	${11.92\pm 0.15}^{\text{a,b,c}}$	${22.33\pm 0.93}^{\text{a,b,c}}$	${62.78\pm 0.27}^{\text{a,b,c}}$	${35.92\pm 0.28}^{\text{a,b,c}}$	1.30±0.63

${}^{\text{a}}$ P<.05 versus normal.

${}^{\text{b}}$ P<.05 versus control mice.

${}^{\text{c}}$ P<.05 versus cisplatin.

**Table 8:** Antimicrobial screening data of ligands and their complexes.

Compound	Representation zone of inhibition		
	Antibacterial	Antifungal	
	B.c	A.n	C.a
ligand	+	++	++
[Cu(L)Cl]⋅2H_2_O	++	++	++
[Cu(L)OAc]⋅2H_2_O	+	++	++
[Ni(L)Cl]	−	+++	++
[Ni(L)OAc]	++	++	++
[Co(L_2_)]	++	+++	+++
[Zn(L_2_)]⋅2H_2_O	+	++	++

Bacillus Cirroflagellosus = B.c.

Aspergillus niger = A.n.

Candida albican = C.a.

10 mm = − (inactive).

10–20 mm = + (weakly active).

21–25 mm = ++ (moderatively active).

26–35 mm = +++ (highly active).

36–40 mm = ++++ (most active).

DMF = 12 mm.

Norfloxacin = 29 P.a and 31 B.c;

Grisofulvin = 20 A.n and 23 C.a.

**Index**

(1) Concentration of the compound: 1 mg/ml in dimethyl formamide.

(2) Quantity in each cup: 0.1 ml.

(3) Diameter of the cup: 10 mm.

(4) Control of the antibacterial activity: Norfloxaicn.

(5) Control of the antifungal activity: Grisofulvin.

(6) Solvent used: Dimethyl formamide.
